# Nodule de Sœur Marie-Josèphe révélateur de carcinomes digestif et ovarien: à propos de 4 cas

**DOI:** 10.11604/pamj.2015.22.269.8022

**Published:** 2015-11-20

**Authors:** Papa Souleymane Touré, Cheikh Tidiane Tall, Pauline Dioussé, Adama Berthé, Madoky Maguatte Diop, Mamadou Moustapha Sarr, Balla Diop, Yakham Mohamed Léye, Bernard Marcel Diop, Mamadou Mourtalla Ka

**Affiliations:** 1 Service de Médecine Interne Hôpital Tivaouane, Thiès, Sénégal; 2 UFR des Sciences de la Santé, Université de Thiès, Sénégal; 3 Service de Médecine Interne Centre Hospitalier National de Pikine, Dakar, Sénégal

**Keywords:** Nodules de Sœur Marie-Josèphe, métastase ombilicale, carcinome, Sister Mary Joseph nodule, umbilical metastasis, carcinoma

## Abstract

Le nodule de Sœur Marie-Josèphe est une métastase ombilicale d'une tumeur le plus souvent intra-abdominale. C'est un signe clinique rare dont l'incidence est de 1-3% de toutes les néoplasies abdomino-pelviennes, avec un pronostic péjoratif du fait de son retard diagnostique. Nous rapportons quatre observations d'une métastase cutanée ombilicale révélatrice d'un adénocarcinome dont deux pancréatiques, un gastrique et un d'origine ovarienne. Le but de notre travail est de montrer à travers ces quatre cas cliniques, l'intérêt de l'imagerie (tomodensitométrie, échographie) et de la biopsie dans la démarche diagnostique. A travers ces quatre observations nous insistons aussi sur les difficultés diagnostiques et thérapeutiques que pose cette tumeur dans nos pays à ressources limitées.

## Introduction

L'ombilic peut être le siège d'une lésion tumorale bénigne ou maligne. Lorsqu'elle est maligne la lésion tumorale peut être primaire ou métastatique. Décrite pour la première fois par Sœur Marie-Josèphe en 1928, qui avait identifié la relation entre nodules ombilicaux et cancers intra-abdominaux avancés; l’éponyme «nodule de Sœur Marie-Josèphe» a été utilisé la première fois par Sir Hamilton Bailey, en 1949 [1] afin de décrire l'entité de lésions métastatiques ombilicaux. Le nodule de Sœur Marie Josèphe (SMJ) est un signe clinique discret et rare, qui ne montre pas seulement la présence d'une tumeur maligne viscérale mais révèle également le mauvais pronostic de ces cancers [2]. Il pose un problème de diagnostic étiologique car la recherche de la tumeur primitive n'est pas toujours aisée.Le but de notre travail était de mettre en exergue le retard diagnostique, à travers quatre observations de nodule de SMJ révélateur d'adénocarcinome digestif et ovarien.

## Patient et observation

### Observation 1

**Figure 1:**
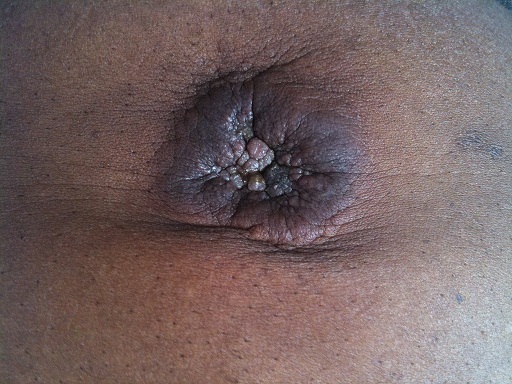
Nodule de Sœur Marie-Josèpheau cours d'un adénocarcinome corporéo-isthmique du pancréas

Patient âgé de 75 ans hospitalisé en août 2010 pour une tuméfaction ombilicale douloureuse prurigineuse apparue depuis 3 mois; dans un contexte de douleurs abdominales, d'amaigrissement évoluant depuis 5 mois. L'examen montrait hormis une altération de l’état général, un nodule ombilical arrondi à contours irréguliers, ulcéré, mesurant 7 cm x 5 cm (Figure 1). Le dosage des marqueurs tumoraux montrait une élévation du CA19-9 à 532 U/ml (N < 37 U/ml) et de l'ACE à 14ng/ml (N< à 5 ng/ml). Le taux d'alpha fœtoprotéine était normal. La tomodensitométrie (TDM) abdominale dévoilait une masse tumorale du pancréas corporéo-isthmique, de 69 mm de diamètre, associée à de multiples nodules hépatiques d'allure secondaire et à de nombreuses adénopathies cœlio-mésentériques. La cytoponction du nodule était en faveur d'un adénocarcinome du pancréas. Ce diagnostic avec métastases ombilicale et hépatique était retenu. Un traitement palliatif antalgique était prescrit. Le décès survenait deux mois plus tard.

### Observation 2

**Figure 2:**
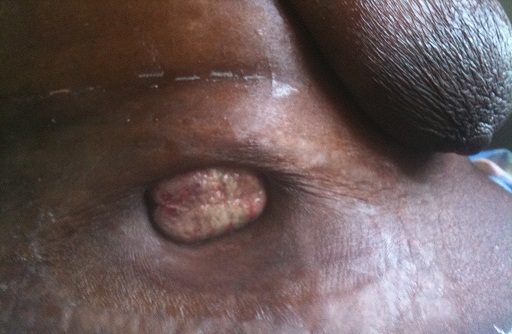
Nodule de Sœur Marie-Josèphe au cours d'un cancer de l'ovaire

Une femme de 68 ans, sans antécédent particulier, était admise en fevrier 2011 pour l'apparition depuis 4 mois d'une tuméfaction ombilicale secondairement ulcérée, suintante. Ce tableau était associé à des douleurs abdominales diffuses avec augmentation du volume de l'abdomen sans notion de trouble du transit. Il n’était pas noté des métrorragies. L'examen montrait une altération de l’état général à 4 selon l’échelle de l'OMS, un nodule ulcéré de l'ombilic. Ce dernier était suintant, douloureux, mesurant 6 cm de diamètre dans son plus grand axe (Figure 2). Par ailleurs, il existait une ascite libre de moyenne abondance, une hépatomégalie d'allure tumorale. Les touchers pelviens étaient normaux. L'examen cytologique de l'ascite montrait la présence de cellules malignes. Le CA 125 était élevé à 38 ng/ml (N< à 5 ng/ml). L’échographie abdominale mettait en évidence une masse latéro-utérine droite de 84 mm en accord avec une tumeur ovarienne, une hépatomégalie hétéro-multinodulaire d'allure secondaire et une ascite de moyenne abondance. Le diagnostic le plus probable était un cancer de l'ovaire droit avec métastases ombilicale, péritonéale et hépatique. La prise en charge se limitait à un traitement symptomatique. Le décès est survenu 15 jours après son admission.

### Observation 3

**Figure 3:**
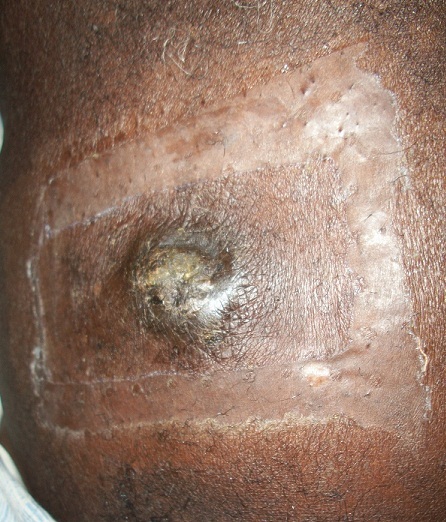
Nodule de Sœur Marie-Josèphe au cours d'un adénocarcinome de la queue du pancréas

Il s'agissait d'un patient de 78 ans, vu en consultation en novembre 2012 pour une masse ombilicale douloureuse ulcérée suintante prurigineuse (Figure 3); sur un fond de constipation opiniâtre. Il était suivi 4 mois auparavant pour un ulcère antral bénin à l'histologie, révélé par une hématémèse mal tolérée. Le patient ne présentait aucun autre signe clinique. La biopsie de la masse ombilicale a révélé la présence d'un adénocarcinome bien differencié. La recherche étiologique mettait en évidence à la colonoscopie un aspect de compression extrinsèque de l'angle colique gauche; dont la TDM retrouvait une tumeur de la queue du pancréas associée à un nodule de la petite courbure gastrique. L’évolution été marquée par l'apparition en quelque semaines d'une ascite carcinomateuse. Le patient décéda 8 mois après ce diagnostic d'un adénocarcinome du pancréas métastatique.

### Observation 4

**Figure 4:**
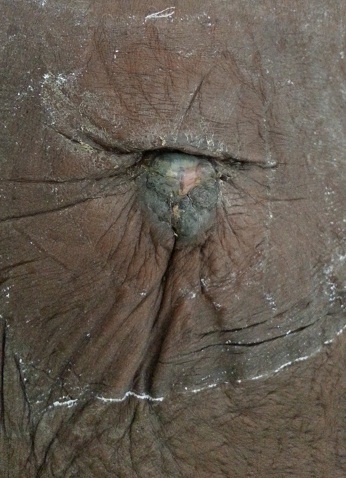
Nodule de Sœur Marie-Josèphe au cours d'un adénocarcinome gastrique

Une patiente de 61 ans était reçue en juillet 2014 pour une ulcération chronique de 2cm de l'ombilic (Figure 4), avec des douleurs abdominales, des vomissements chroniques, une candidose buccale; dans un contexte d'altération de l’état général évoluant depuis 2 mois. L'examen gynécologique ne révélait pas de notion de métrorragie. Le col et les parois vaginales étaient macroscopiquement sains. La TDM abdomino-pelvienne mettait en évidence un épaississement circonférentiel de l'antre gastrique ainsi qu'un nodule tissulaire ombilical de 2 cm. L'endoscopie oeso-gastro-duodénale révélait une tumeur ulcèrobourgeonnante sténosante médiogastrique d'environ 5 cm, dont l'histologie n'a pu être effectuée. La biopsie ombilicale concluait à une image histologique et immuno-histochimique compatible avec un adénocarcinome à point de départ digestif. La patiente décéda 2 mois après le diagnostic.

## Discussion

Le nodule de SMJ est un nodule palpable de l'ombilic secondaire le plus souvent à une métastase d'un cancer abdomino-pelvien [3]. C'est un signe clinique discret et rare. Son incidence est faible, puisqu'on estime que 1-3% des patients atteints de néoplasies abdomino-pelviennes pourrait présenter un nodule de SMJ [4]. Dans 30%, les nodules de SMJ sont le mode de présentation initiale, voire le seul signe d'une néoplasie. Dans la moitié de ces présentations initiales par nodule de SMJ, la lésion primaire demeurera occulte. Cette métastase ombilicale peut également représenter un signe de récidive ou de progression néoplasique, souvent vers la carcinose péritonéale [3, 5]. Le tractus gastro-intestinal est la localisation la plus fréquente de la tumeur primaire (35 à 65%), suivie d'une origine gynécologique (12 à 35%). D'autres origines extra péritonéales sont rapportées; tel que le poumon, le sein et le pénis dans 3%-6%. Les origines digestives les plus rapportées par ordre décroissant sont: l'estomac (25%), le colon ou le rectum (10%) et le pancréas (7%) [3, 6]. L'aspect clinique du nodule de SMJ est souvent celle d'une tuméfaction généralement ferme, indurée avec des marges irrégulières. La surface est parfois fissurée ou ulcéro-nécrotique; souvent douloureuse voir prurigineuse. La taille est variable, de 0,5 à 2 cm en général. Quelques cas spectaculaires jusqu’à 10 cm ont été rapportés. Selon l'origine histologique, il peut existait un écoulement séreux, sanguin, mucoide ou purulent [3, 7]. De multiples lésions peuvent entrer dans le diagnostic différentiel des masses ou épaississements ombilicaux. On trouve notamment les causes bénignes telles que: l'endométriose, le naevus mélanocytaire, le dermatofibrome, le kyste de l'ouraque, la kératose séborrhéique, le kyste pilonidal, le granulome de talc, le granulome pyégénique, l'omphalite, la chéloïde, le corps étranger, l'abcès, voir l'hernie [8]. Les tumeurs primitives ombilicales quand à elles sont beaucoup plus rares. Différents types de tumeurs ont été rapportés tels que: le mélanome, le carcinome baso-cellulaire, le carcinome épidermoïde, le myosarcome, l'adénocarcinome primitif [9]. L'imagerie est insuffisante pour distinguer ces lésions bénignes ou malignes les unes des autres. La biopsie, souvent facile à réaliser sans ou avec guidance échographique, est alors l'investigation de choix pour étayer le diagnostic. Plusieurs mécanismes physiopathologiques sont avancés pour expliquer la survenue du nodule de SMJ. Etant donné l’étroite proximité avec le péritoine, l'extension directe de lésions péritonéales reste le mécanisme physiopathologique le plus fréquent des métastases ombilicales. D'autres mécanismes sont évoqués, comme une dissémination hématogène à travers les systèmes artériels et veineux, une extension lymphatique (carcinome du pancréas principalement), une extension le long des ligaments d'origine embryonnaire (ligament rond de foie, l'ouraque, le vestige du conduit vitello-intestinal). En outre, l'implantation directe suite à une laparoscopie est un autre mode de propagation des tumeurs à l'ombilic [6, 8]. La présence de nodules de SMJ signifie habituellement, un cancer métastasé avancé, de mauvais pronostic. La découverte d'un nodule métastatique sur le site ombilical établit presque certainement le caractère non opérable du patient [8]. Dans nos pays à ressources limitées, l'exploration d'un nodule de SMJ pose des défis diagnostiques et thérapeutiques majeurs. Le diagnostic tardif dans notre étude peut être attribuée en partie au caractère discret du nodule de SMJ souvent banalisé par les patients. Il s'y ajoute une méconnaissance de la maladie par les praticiens, le manque d'accessibilité aux structures sanitaires de référence, et l'absence des aides au diagnostic de pointe telles que la tomodensitométrie, les marqueurs tumoraux et l'anatomopathologie dans nos structures sanitaires. Ce diagnostic tardif à un stade avancé comme chez tous les 4 patients de notre étude, couplé à l'inaccessibilité des schémas thérapeutiques tels que la chimiothérapie adjuvante; sont ainsi des obstacles à une prise en charge optimale de ces malades dans les pays en développement.

## Conclusion

Le nodule de SMJ reste encore une tumeur rare d'origine métastatique d'un cancer le plus souvent digestif. Le pronostic encore très sombre, impose un dépistage précoce donc systématique. Ceci passe par une biopsie de tout nodule ou masse ombilicaux pour déterminer la nature de la lésion pathologique.

## References

[1] Trebing D, Göring HD (2004). The umbilical metastasis. Sister Mary Joseph and her time. Hautarzt..

[2] Majmudar B, Wiskind AK, Croft BN, Dudley AG (1991). The Sister (Mary) Joseph nodule: its significance in gynecology. Gynecol Oncol..

[3] Gabriele R, Conte M, Egidi F, Borghese M (2005). Umbilical metastases: current viewpoint. World J Surg Oncol..

[4] Dubreuil A, Dompmartin A, Barjot P, Louvet S, Leroy D (1998). Umbilical metastasis or Sister Mary Joseph's nodule. Int J Dermatol..

[5] Papalas JA, Selim MA (2011). Metastatic vs primary malignant neoplasms affecting the umbilicus: clinicopathologic features of 77 tumors. Ann Diagn Pathol..

[6] Al-Mashat F, Sibiany AM (2010). Sister Mary Joseph's nodule of the umbilicus: is it always of gastric origin? A review of eight cases at different sites of origin. Indian J Cancer..

[7] Palaniappan M, Jose W M, Mehta A, Kumar K, Pavithran K (2010). Umbilical metastasis: a case series of four Sister Joseph nodules from four different visceral malignancies. Curr Oncol..

[8] Chalya PL, Mabula JB, Rambau PF, McHembe MD (2013). Sister Mary Joseph's nodule at a University teaching hospital in northwestern Tanzania: a retrospective review of 34 cases. World J Surg Oncol..

[9] Kluger N (2014). Dermatoses ombilicales et péri-ombilicales. Ann Dermatol Venereol.

